# Impact of gender on response to highly active antiretroviral therapy in HIV-1 infected patients: a nationwide population-based cohort study

**DOI:** 10.1186/1471-2334-12-293

**Published:** 2012-11-12

**Authors:** Kristina Thorsteinsson, Steen Ladelund, Søren Jensen-Fangel, Isik Somuncu Johansen, Terese L Katzenstein, Gitte Pedersen, Merete Storgaard, Niels Obel, Anne-Mette Lebech

**Affiliations:** 1 Department of Infectious Diseases, Hvidovre, Copenhagen University Hospital, Kettegaards Allé 30, Hvidovre DK-2650, Denmark; 2 Clinical Research Center, Hvidovre, Copenhagen University Hospital, Copenhagen, Denmark; 3 Department of Infectious Diseases, Skejby, Aarhus University Hospital, Aarhus, Denmark; 4 Department of Infectious Diseases, Odense University Hospital, Odense, Denmark; 5 Department of Infectious Diseases, The National University Hospital, Rigshospitalet, Copenhagen, Denmark; 6 Department of Infectious Diseases, Aalborg University Hospital, Aalborg, Denmark

**Keywords:** HIV, Gender differences, Modification, HAART, Viral suppression

## Abstract

**Background:**

Impact of gender on time to initiation, response to and risk of modification of highly active antiretroviral therapy (HAART) in HIV-1 infected individuals is still controversial.

**Methods:**

From a nationwide cohort of Danish HIV infected individuals we identified all heterosexually infected women (N=587) and heterosexually infected men (N=583) with no record of Hepatitis C infection diagnosed with HIV after 1 January 1997. Among these subjects, 473 women (81%) and 435 men (75%) initiated HAART from 1 January 1997 to 31 December 2009. We used Cox regression to calculate hazard ratio (HR) for time to initiation of HAART, Poisson regression to assess incidence rate ratios (IRR) of risk of treatment modification the first year, logistic regression to estimate differences in the proportion with an undetectable viral load, and linear regression to detect differences in CD4 count at year 1, 3 and 6 after start of HAART.

**Results:**

At initiation of HAART, women were younger, predominantly of Black ethnicity and had a higher CD4 count (adjusted *p*=0.026) and lower viral load (adjusted *p*=0.0003). When repeating the analysis excluding pregnant women no difference was seen in CD4 counts (adjusted *p*=0.21). We observed no delay in time to initiation of HAART in women compared to men (HR 0.91, 95% CI 0.79-1.06). There were no gender differences in risk of treatment modification of the original HAART regimen during the first year of therapy for either toxicity (IRR 0.97 95% CI 0.66-1.44) or other/unknown reasons (IRR 1.18 95% CI 0.76-1.82). Finally, CD4 counts and the risk of having a detectable viral load at 1, 3 and 6 years did not differ between genders.

**Conclusions:**

In a setting with free access to healthcare and HAART, gender does neither affect time from eligibility to HAART, modification of therapy nor virological and immunological response to HAART. Differences observed between genders are mainly attributable to initiation of HAART in pregnant women.

## Background

Since the introduction of highly active antiretroviral therapy (HAART) gender differences on HIV therapies have been reported including (i) time of HAART initiation [1], (ii) adherence and toxicity to antiretroviral drugs [2-4], and (iii) virological and immunological response to HAART [5,6] .

Despite the reported gender differences and differences in circulating blood volume and body weight, current treatment goals and dosage of antiretrovirals are equivalent in HIV-1 (HIV) positive women and men [7]. One of the reasons for this may be that women are underrepresented in many HIV clinical trials. In fact, according to a large metaanalysis of 43 randomized clinical trials from 2000–2008 women only accounted for 20% of 22,411 HIV positive subjects [8].

A reduced tolerability of antiretroviral drugs in women compared to men has been reported [2]. Poor tolerability can affect adherence and an association between female gender and reduced rate of adherence to HAART has been described [3,4], but findings are inconsistent and depends on the composition of the studied cohort [4].

Most women with HIV in Europe and the US are of childbearing age and the intention for childbearing is high in this population [9,10]. Thus when prescribing HAART one must consider pregnancy and avoid drugs that are not recommended for early antenatal use e.g. efavirenz and didanosine.

Because of the significant reduction in mortality and rate of disease progression following HAART [11,12], surrogate markers of disease progression such as viral load and CD4 count have been introduced [13,14].

Most studies report no gender-related differences in terms of virological and immunological response to HAART, however data are conflicting [2,6,8,15].

In the present study we used a nationwide, population based cohort of heterosexually infected individuals to estimate gender differences in initiation of HAART regarding timing, regimen and modifications. Moreover, we aimed to estimate the effectiveness of HAART by means of viral load and CD4 count in genders in a setting with free access to HAART and healthcare.

## Methods

### Setting

Denmark has a population size of 5.6 million [16] and an estimated HIV prevalence among adults of 0.1% [17]. Medical care, including HAART, is tax-paid and provided free-of-charge to all HIV-infected residents in Denmark. Treatment of HIV-infected patients is restricted to eight specialized medical centres, where patients are seen on an outpatient basis at intended intervals of 12–24 weeks. National criteria for initiation of HAART are: (i) acute HIV-infection, (ii) HIV-related disease or an AIDS defining illness (ADI), (iii) pregnancy, (iv) until 1 May 2008 a CD4 count below 300 cells/μl and hereafter a CD4 count below 350 cells/μl and (v) until 31 December 2001 HIV-1 RNA > 100,000 copies/mL [18].

### The Civil Registration System (CRS)

The CRS is a national registry of all Danish residents containing information on date of birth, sex, address, date of migration and date of death. At birth or immigration a 10-digit personal number is assigned to each individual (CPR), which enables treatment centres to avoid multiple registrations of the same patient. We used the CPR to link data from the CRS to the Danish HIV Cohort Study.

### Danish HIV Cohort Study (DHCS)

The DHCS is a prospective, observational, nationwide, multicentre population-based cohort study of all HIV-infected patients seen at the Danish HIV clinics since 1 January 1995. The cohort has been described in details elsewhere [19]. In brief, the data collection is ongoing, with continuous enrolment of both newly diagnosed residents and immigrants with HIV. The database is updated annually and among other variables it contains data on: date of birth, gender, route of transmission, race, date of first HIV-1 positive test, bodyweight, immigration and emigration, date of death, date of HAART initiation, HAART regimens and reasons for modifications. Laboratory data include cumulative CD4 counts and HIV RNA among others. Status of pregnancy was extracted from the Danish National Hospital Database.

### Ethics

The study has been approved by the Danish Data Protection Agency (jr. 2001-41-0624).

The DHCS is approved by the Danish Data Protection Agency. Since data collection did not involve direct patient contact the study was not subject to approval by the Danish Research Ethics Committee.

### Study population

We describe two cohorts in this study: Cohort 1: Heterosexually infected adult women and men diagnosed with HIV in Denmark from 1 January 1997 to 31 December 2009 with no record of Hepatitis C (HCV) infection. Cohort 2: Patients in the above mentioned cohort initiating HAART from 1 January 1997 to 31 December 2009.

### Definitions

HAART was defined as the combination of antiretroviral treatment with at least three drugs, including at least one non-nucleoside reverse-transcriptase inhibitor (NNRTI) or a protease inhibitor (PI), and/or abacavir, or a treatment regimen with a combination of a NNRTI and a boosted PI [18]. We categorized treatment modifications into 3 groups: virological failure, toxicity (covering: abnormalities of body fat distribution, dyslipidaemia, hypersensitivity, gastrointestinal toxicity, neurological toxicity, nephrological toxicity, endocrinological toxicity and other toxicity) and other/unknown (covering: patient’s wish, doctor’s decision, other cause, unknown, as part of modification to another HAART, problems with adherence). Data were based on medical files.

Virological failure was defined as such if the physician treating the patients’ HIV infection had stated virological failure as the reason for modification in the medical file.

Undetectable viral load was defined as a plasma HIV RNA load of <500 copies/mL, which was the highest level of sensitivity for testing in the observation period. We defined acute HIV infection as clinical seroconversion with a positive Western blot pattern.

CD4 count and HIV RNA at HAART initiation were defined as the latest value measured before initiation of therapy. Hepatitis B (HBV) co-infection was defined as being hepatitis B surface antigen (HbsAg) positive.

### Statistical analyses

Start of the observation period for cohort 1 was defined as date of eligibility for therapy as described above and patients were followed to initiation of HAART, emigration, death or 31 December 2009, whichever came first. Cohort 1 is used in the analysis of time from eligibility for therapy to HAART initiation.

In cohort 2 patients were observed from date of HAART initiation and censored at emigration, death or 31 December 2009, whichever came first. Cohort 2 was studied in all analyses, but timing of HAART initiation.

A patient was considered eligible for therapy the day they fulfilled one of the above mentioned national criteria concerning CD4 count, HIV RNA or AIDS.

The incentive for initiation of HAART during pregnancy, acute HIV infection and in HBV coinfection is different than in patients starting HAART due to an impaired immune system, i.e. low CD4 count. In the analysis of time from eligibility to HAART initiation we therefore excluded patients initiating HAART before they were eligible for therapy.

Information on initiation of HAART due to non-AIDS defining HIV related disease was not available in the DHCS and therefore not assessed in the analysis of being eligible for start of HAART. When studying the actual number of patients initiating HAART during acute HIV infection this was defined as a patient with acute HIV infection initiating therapy during the first 3 months after HIV diagnosis.

Intergroup characteristics were compared using Wilcoxon rank sum test for continuous variables and chi-square test and Fisher’s exact test for categorical variables. Median and interquartile ranges (IQR) were determined for continuous variables. The CD4 count at initiation of HAART was subsequently log-transformed and analysed in linear regression adjusting for age, ethnicity and period of initiation. AIDS was not included in this analysis as covariate, since the CD4 count and AIDS are dependent covariates. Likewise, viral load at initiation of HAART was log-transformed and analysed in linear regression adjusting for age, ethnicity, period of initiation and prior or current AIDS at start of HAART.

To assess linearity, continuous variables were entered in the model as second degree polynomials and reduced as appropriate. Significance level was set at 0.05 (two-sided).

To evaluate the response to HAART, the CD4 and HIV RNA values were grouped in 12-week intervals and computed as described elsewhere [20]. The absolute CD4 counts were log-transformed and analysed in linear regression and adjusted as mentioned above at 1 year (48 weeks), 3 years (156 weeks) and 6 years (312 weeks). Furthermore, we estimated the proportion of patients achieving an undetectable viral load during follow-up. Logistic regression model was used to compare prevalence of detectable viral load between genders at year 1, 3 and 6. Odds ratios (OR’s) and correlating 95% confidence intervals (CI) were estimated and adjusted for age, ethnicity, period of initiation and prior or current AIDS at initiation of HAART. The validity of the model was tested using the Hosmer and Lemeshow Goodness-of-Fit Test.

The cumulative incidence function with death as a competing risk were computed for time from eligibility of therapy to HAART initiation. To estimate the hazard ratio (HR) and associated 95% CI, we used Cox proportional hazards models and adjusted for race and time-updated age (18–29, 30–39, 40–49, 50–59, 60–69 and 70–100 years). Due to non-proportionality of period of initiation the analysis was stratified for period of initiation of HAART.

In the sensitivity analyses women initiating HAART due to pregnancy, women were excluded if date of HAART initiation was within the period of one year before the conception (estimated as 37 weeks before delivery) or during pregnancy.

Two periods: 1997–2002 (the early HAART period) and 2003–2009 (the late HAART period) and five initial HAART regimens were assessed for analysis in this study: i) 3 nucleoside reverse transcriptase inhibitors (NRTI’s), ii) 2 NRTI’s + efavirenz, iii) 2 NRTI’s + nevirapine, iv) 2 NRTI’s + PI/ritonavir or PI and v) other HAART regimen.

Poisson regression analyses were used to estimate IR, IRR and corresponding 95% CI of the first modification of treatment due to toxicity and other/unknown the first year after initiation of HAART and were adjusted by time-updated age (18–29, 30–39, 40–49, 50–59, 60–69, 70–100 years), race, time-updated CD4 count (<200, 200–350 and >350 cells/μl), time-updated viral load (< 50,000, 50,000-100,000 and > 100,000 copies/mL), prior or current AIDS at HAART initiation, body weight (<50, 50–70, 71–100 and >100 kg), initiation period < 1 January 2003 and >= 1 January 2003. Modification due to failure could not be assessed because of small numbers.

A composite table was computed with reasons for switching due to toxicity. In these analyses a patient could only contribute with one modification of each reason. In each category with more than five changes of regimen IR’s and corresponding 95%CI and IRR’s and corresponding 95%CI adjusted for the same confounders as mentioned above in the analyses of IRR.

The cumulative incidence function of time from eligibility to HAART initiation were done using R-2.12.2 [21] and the cmprsk library by Bob Gray.

SAS statistical software version 9.2 (SAS Institute Inc., Cary, NC, USA) was used for data analysis.

## Results

### Time from eligibility to HAART initiation

**Figure 1:**
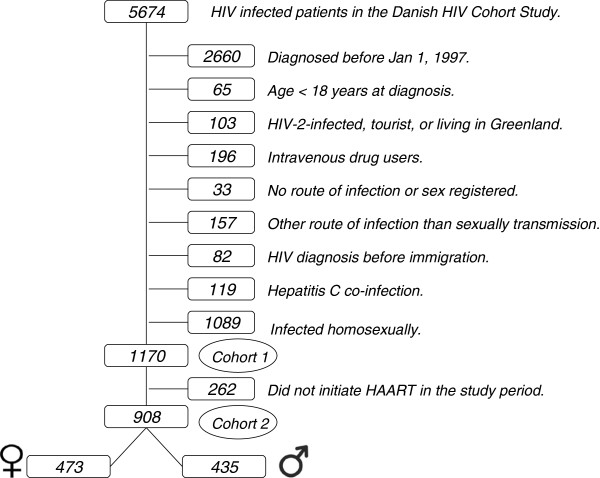
Flowchart of HIV infected patients in the Danish HIV Cohort Study.

**Figure 2:**
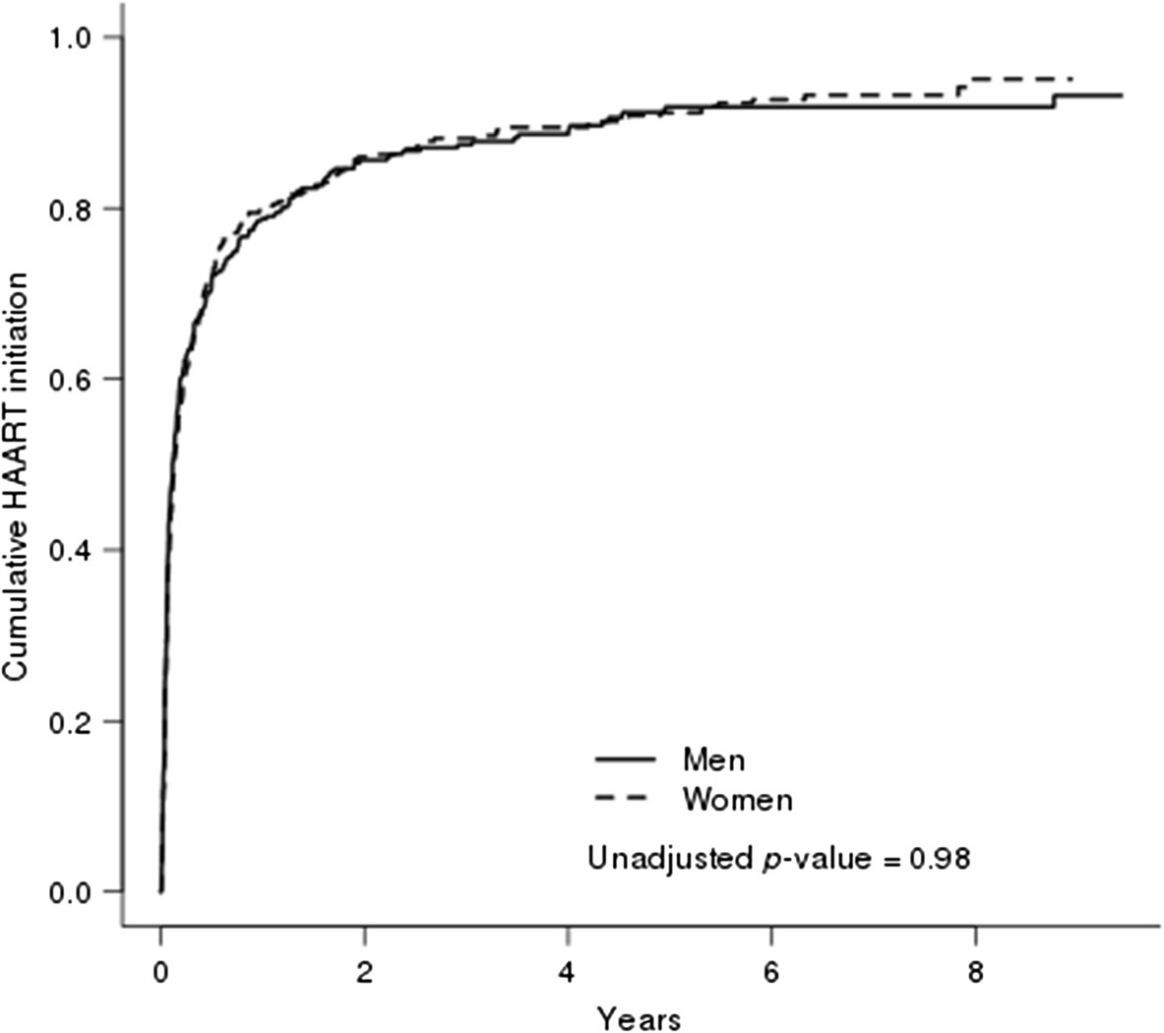
The cumulative incidence function with death as a competing risk for time from eligibility of therapy to HAART initiation stratified by gender.

A total of 1170 heterosexually infected women and men reached eligibility for HAART. Subsequently, HAART was initiated in 473 (52.1%) women and in 435 (47.9%) men during follow-up (Figure 1). Figure 2 presents curves for time from eligibility to initiation of HAART stratified by gender. We found no difference in time to initiation of HAART between genders (HR 0.91 95% CI, 0.79-1.06, unadjusted *p*=0.98, adjusted *p*=0.21 (women vs. men).

### Patient characteristics

Clinical and demographic characteristics at initiation of HAART are presented in Table 1. Total follow-up: 5352 person-years. Women were younger, a greater proportion of women were of Black and Asian ethnicity (*p*<0.0001) and had acquired HIV abroad (*p*<0.0001). Women initiated HAART at higher CD4 counts (adjusted *p*=0.026) and lower viral loads (adjusted *p*=0.0003). Fifty-nine women (12.5%) initiated HAART due to pregnancy. When repeating the analysis excluding women who initiated HAART due to pregnancy there was no difference in median CD4 counts at initiation of HAART (adjusted *p*=0.21). Prior or current AIDS and acute HIV infection at HAART initiation were more frequent in men. During the late HAART era women were more likely to receive a HAART regimen comprising 2 NRTI’s + either PI/r or unboosted PI (29.1% vs. 9.0%), and less likely to initiate a 2 NRTI’s + efavirenz based regimen (59.8% vs. 80.3%) than men (*p*<0.0001). This difference was not significant when women initiating HAART due to pregnancy were excluded.

**Table 1:** Patient characteristics at initiation of HAART stratified by gender (n = 908)

	**Women**	**Men**	***p-value***^***1***^
Subjects, n(%)	473 (52.1)	435 (47.9)	-
Follow-up from initiation of HAART (years), median (IQR)	5.9 (3.2-8.7)	5.7 (2.7-8.5)	-
Follow-up time from initiation of HAART, (person-years)	2833	2519	
Age in years at HAART initiation, median (IQR)	33.0 (28.6-40.1)	43.2 (35.3-51.7)	<0.0001
Race, n(%)			
White	137 (29.0)	319 (73.7)	<0.0001
Asian	76 (16.1)	12 (2.8)	
Black	250 (53.0)	90 (20.8)	
Other	9 (1.9)	12 (2.8)	
(missing)	(1)	(2)	
Place of HIV transmission, n(%)			
Denmark	127 (28.5)	150 (38.3)	<0.0001
Europe + US	19 (4.3)	29 (7.4)	
Africa	224 (50.3)	140 (35.7)	
Asia	72 (16.2)	63 (16.1)	
Other	3 (0.7)	10 (2.6)	
(missing)	(28)	(43)	
CD4 cell count at HAART initiation (cells/μl), median (IQR)			
All women included	196 (90–290)	180 (63–290)	0.12 (0.026)
Pregnant women excluded	170 (80–270)	180 (63–290)	0,87 (0,21)
HIV-RNA at HAART initiation (copies/mL), median (IQR) All women included	50,800 (12,500-204,000)	126,155 (32,200-501,000)	<0.0001 (0.0003)
Pregnant women excluded	61,320 (16,519-237,000)	126,155 (32,200-501,000)	<0.0001 (0.0066)
Acute HIV infection at HAART initiation, n(%)	4 (0.9)	15 (3.5)	0.0089
AIDS before HAART initiation, n(%) All women included	88 (18.6)	106 (24.4)	0.034
n(%) of all patients except pregnant women Pregnant women excluded	87 (21.8)	106 (24.4)	0.37
Pregnant at HAART initiation, n(%)	59 (12.5)	-	-
Antiretroviral therapy before HAART, n(%)	14 (3.0)	12 (2.8)	0.86
Hepatitis B co-infection^2^, n(%)	30 (6.3)	20 (4.6)	0.25
Hepatitis C co-infection^3^, n(%)	0 (0)	-	-
First-line HAART 01.01.1997 – 31.12.2002 All women included			
3 NRTI’s^4^	21 (9.5)	9 (4.3)	0.29
2 NRTI’s + efavirenz	71 (32.0)	76 (35.9)	
2 NRTI’s + nevirapine	7 (3.2)	6 (2.8)	
2 NRTI’s + PI’s^5^	93 (41.9)	94 (44.3)	
Other regimen	30 (13.5)	27 (12.7)	
Pregnant women excluded			
3 NRTI’s	21 (11.1)	9 (4.7)	0.13
2 NRTI’s + efavirenz	66 (37.7)	76 (39.8)	
2 NRTI’s + nevirapine	5 (2.6)	6 (3.1)	
2 NRTI’s + PI’s	73 (38.4)	94 (44.3)	
Other regimen	25 (13.2)	27 (12.7)	
First-line HAART 01.01.2003 – 31.12.2009 All women included			
3 NRTI’s	3 (1.2)	1 (0.5)	<0.0001
2 NRTI’s + efavirenz	150 (59.8)	179 (80.3)	
2 NRTI’s + nevirapine	17 (6.8)	13 (5.8)	
2 NRTI’s + PI’s	73 (29.1)	20 (9.0)	
Other regimen	8 (3.2)	10 (4.5)	
Pregnant women excluded			
3 NRTI’s	3 (1.4)	1 (0.5)	0.066
2 NRTI’s + efavirenz	148 (70.5)	179 (80.3)	
2 NRTI’s + nevirapine	15 (7.1)	13 (5.8)	
2 NRTI’s + PI’s	36 (17.1)	20 (9.0)	
Other regimen	8 (3.8)	10 (4.5)	

Figures are number (%) or median (interquartile range - IQR).

^1^*P*-values were calculated using chi-square or Fisher’s exact test (when appropriate) for categorical variables and Kruskal-Wallis test for continuous variables. CD4 cell counts and viral loads were subsequently analysed by linear regression adjusting for age and race (*p*-value in parenthesis). ^2^ Patients with positive Hbs-Ag in the study period, ^3^ Hepatitis C was an exclusion criteria, ^4^ Nucleoside reverse transcriptase inhibitors (NRTI), ^5^ Protease Inhibitors (PI’s).

### Modification of HAART

During the first year after initiation of HAART, gender had no impact on risk of first modification of treatment due to either toxicity (adjusted incidence rate ratio women vs. men, IRR, 0.97 95% confidence interval (CI) 0.66-1.44) or other/unknown reasons (adjusted IRR women vs. men 1.18 95% CI, 0.76-1.85) (Table 2). Nor did the estimates change when we excluded bodyweight (data not shown). Only 5 (1.3%) women and 4 (1.5%) men switched HAART regimen because of virological failure and therefore this aspect was not further explored.

**Table 2:** Incidence rates, IR (per 1 person-year), incidence rates ratios, IRR, of reasons of treatment modifications the first year after initiation of HAART according to gender

	**Toxicity IR (95% CI)**	***Other/Unknown *IR (95% CI)**	**Toxicity IRR (95% CI)**	***Other/Unknown *IRR (95% CI)**
*Adjusted for all listed confounders,*				
Men	0.52 (0.42-0.64)	0.55 (0.44-0.68)	1.00	1.00
Women	0.49 (0.41-0.59)	0.57 (0.48-0.67)	0.97 (0.66-1.44)	1.18 (0.76-1.85)
Women (pregnant excluded)	0.54 (0.45-0.66)	0.49 (0.40-0.61)	1.03 (0.69-1.52)	1.14 (0.71-1.82)

Poisson regression analyses of incidence rates, IR (95% CI), and incidence rates ratios, IRR (95% CI), adjusted by time-updated age, race, time-updated CD4 count, time-updated viral load, prior or current AIDS at HAART initiation, body weight and initiation period (< January 1^st^ 2003 and >= January 1^st^ 2003).

**Figure 3:**
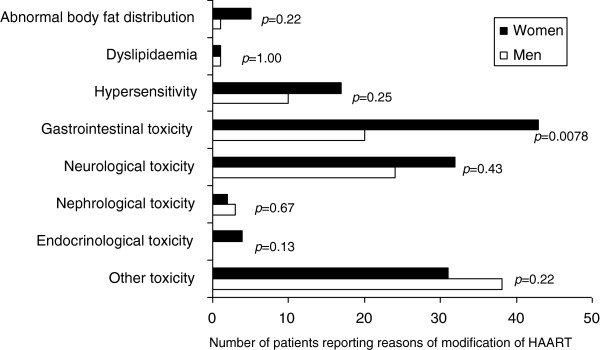
Number of patients reporting modification of HAART during the first year after initiation due to toxicity for the reasons listed.

Overall, the four most common reasons for change of HAART were hypersensitivity, gastrointestinal toxicity, neurological toxicity and other toxicity (Figure 3). Women were more likely to modify their treatment due to gastrointestinal toxicity (9.1% vs. 4.6%, *p*=0.0078). However, the adjusted IRR of risk of switching did not exhibit gender differences (Table 3).

**Table 3:** Incidence rates, IR (per 1 person-year), incidence rates ratios, IRR, of risk of treatment modification due to different types of toxicities

**Incidence rates and incidence rate ratios for changes in HAART during the first year due to the following reasons:**	**Women IR 95%(CI)**	**Men IR 95%(CI)**	**Women vs. men (ref) unadjusted IRR 95%(CI)**	**Women vs. men (ref) adjusted IRR 95%(CI)**
Toxicity, hypersensitivity	0.09 (0.06-0.15)	0.06 (0.03-0.14)	1.43 (0.58-3.52)	−^1^
Toxicity, gastrointestinal	0.22 (0.16-0.15)	0.16 (0.10-0.26)	1.37 (0.80-2.36)	0.97 (0.49-1.93)
Toxicity, neurological	0.19 (0.13-0.27)	0.20 (0.13-0.30)	0.96 (0.56-1.64)	1.48 (0.65-3.38)
Toxicity, other	0.18 (0.13-0.25)	0.29 (0.21-0.40)	0.62 (0.39-1.00)	−^1^

^1^ No estimate available, due to shortage of events.

Poisson regression analyses of incidence rates, IR (95% CI), and incidence rates ratios, IRR (95% CI), adjusted by time-updated age, race, time-updated CD4 count, time-updated viral load, prior or current AIDS at HAART initiation, body weight and initiation period (< January 1st 2003 and >= January 1st 2003).

### Response to HAART

**Figure 4:**
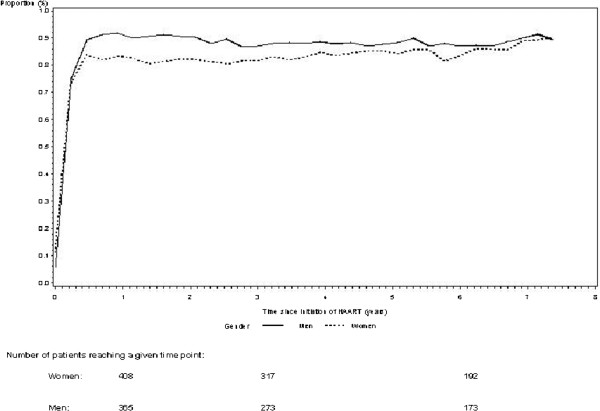
Proportion of patients with an undetectable viral load during the study period.

We estimated the proportion of patients with an undetectable viral load after initiation of HAART (Figure 4). One year after initiation of HAART 83% of women and 92% of men had gained viral control (viral load < 500 copies/ml), adjusted OR 1.24 (95%CI 0.72-2.14). After 3 and 6 years the proportion of women and men with an undetectable viral load was 82% and 87%, respectively, adjusted OR 0.92 (95%CI 0.53-1.61), and 83% and 87%, respectively, adjusted OR 0.87 (95%CI 0.41-1.84)

**Figure 5:**
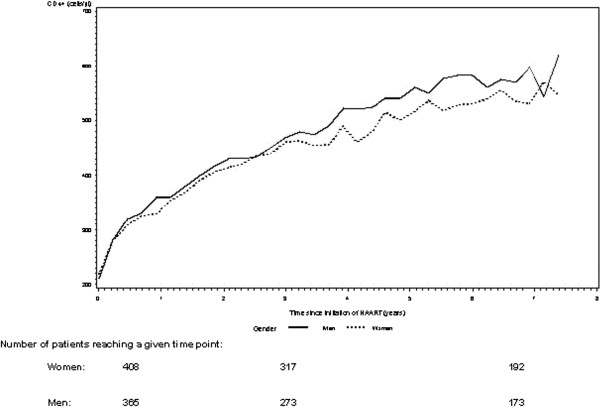
Absolute CD4 count during the study period.

We found no statistically significant differences in median CD4 count between genders after initiation of HAART. At year 1 the median CD4 count was 330 cells/μl IQR(210–457) in women and 360 cells/μl IQR(220–510) in men (adjusted *p*=0.90) at 3 years 459 cells/μl IQR(316–600) in women and 469 cells/μl IQR(318–656) in men (adjusted *p*=0.90) and at 6 years 530 cells/μl IQR(380–696) in women and 582 cells/μl IQR(382–793) in men, (adjusted *p*=0.90) (Figure 5).

## Discussion

Trial data on HIV therapies in women are limited [8] and discrepant results regarding the impact of gender on HAART have been reported [2]. Here, we found that in a setting with free access to healthcare and HAART gender did neither affect time from eligibility to HAART, modification of therapy nor virological and immunological response to HAART.

In line with our study, European cohorts report no gender differences in time from eligibility to initiation of therapy [22,23], while in the US a propensity to delay HAART in women compared to men is seen [24-26]. Though, studies defined eligibility differently the estimated differences could be attributed to the reported inequality in access to HAART and healthcare in the US more than gender itself [27]. In Europe, overall there is equality in access to HAART between genders, though in central and eastern Europe women tends to be favored over men [28].

The enormous benefit of HAART is reflected by few clinical outcomes [12,29] and therefore we assessed clinical progression by means of the virological and immunological response in patients seen after initiation of HAART. Additionally, we have earlier reported that in Denmark no major differences in terms of progression to AIDS and death are seen between men who have sex with men (MSM), heterosexually infected men and heterosexually infected women [30]. Therefore we decided to focus on HIV positive patients infected heterosexually to study patients living in the same social context [6].

We excluded intravenous drug users and HCV coinfected patients, since studies repeatedly report worse clinical outcomes [31-33] and weaker immunological and virological responses in these patients [22,34]. Furthermore, due to concerns about residual confounding when adjusting for IDU in the model, we chose to perform restricted analyses with exclusion of this group of patients.

Data on gender differences in CD4 count recovery after initiation of HAART are discrepant. In agreement with others [6,14,15,22] we found no differences in CD4 recovery. On the contrary several studies report a beneficial immunological outcome in women [5,35-37].

The lower viral load seen in women at initiation of therapy even when we excluded pregnant women is supported by several studies [2,3,15,22,35,38,39] and probably has no influence on disease progression [2]. Compared to other cohorts [5] where HIV RNA was undetectable in 48-79% of all patients a relatively high proportion of our patients (83% of women and 92% of men) had undetectable viral load after 1 year, though, different definitions of viral suppression make studies difficult to compare.

We found no difference in virological response during follow-up between men and women, which is in accordance with earlier reports on gender differences [1,15,22], however during the entire follow-up the proportion of men who had gained full viral control was greater than that of women.

Only a small part of patients were ART experienced before HAART initiation and this proportion did not differ between genders. Along with others [5], we found that women were less likely to receive a HAART regimen containing efavirenz. Since the publication of teratogenicity in animal studies [40] and of neural tube defects in infants exposed to efavirenz in their first trimester [41] guidelines have recommended avoiding this drug in women likely to conceive. Indeed, after excluding women who initiated HAART due to pregnancy from our analysis the initial HAART regimen did not differ significantly between genders.

Regarding pharmacokinetics on HAART, data are limited, but current evidence suggests that gender differences exist [42]. Reportedly, women have been found to be more likely to discontinue ART due to neurological [39] and dermatologic symptoms [3,23,43]. Furthermore, an increased risk of lactic acidosis [44], HIV lipodystrophy [23,45] and hepatic dysfunction [46] have been reported in women. Some have speculated that the higher rate of adverse effects in women are caused by a higher drug exposure in women [35].

Our multivariate model failed to show more treatment modifications in women of the original HAART regimen the first year after initiation compared to men. This finding is supported by others [3,6,8]. Because dosing regimens of HAART are not gender specific, we adjusted for bodyweight in the analysis of IRR for treatment modifications and this did not alter results. Nor exclusion of pregnant women changed results significantly.

In contrast to our study four studies [23,37,47,48] found that women were more likely to discontinue treatment than men. However, the first two studies have not considered pregnancy and in the latter two women were excluded when becoming pregnant. However, the UK CHIC study was able to take pregnancy into account and in this cohort women were more likely to discontinuate treatment for reasons other than virological failure [5].

We found that women were more likely to change regimen due to gastrointestinal toxicity, though, this information needs to be interpreted with care due to small numbers.

As earlier stated some studies report that women are more likely to change regimen, because of HIV lipodystrophy [23,45]. Since we focus on the first year after initiation of HAART only, we probably do not allow for sufficient time for this to be of clinical significance [49].

The major strength of our study is the nationwide population-based design, linking the nationwide registers DHCS and CRS with minor loss to follow-up [50]**]** and access to electronically collected longitudinal data on HIV RNA and CD4 counts.

Some limitations need to be assessed. Data on adherence and socioeconomic status are not available in our cohort and are therefore not assessed for analysis. Information on the study participants was those reported by the providers and then retrospectively summarized for this study. In the DHCS only toxicities leading to a modification are documented and therefore the risk of adverse events might be substantially higher than the incidence reported in our study. Moreover, data on virological failure is based on the discretion of the physician and might therefore not reflect the actual number of virological failures.

The route of transmission is self-reported and therefore can be misclassified. Furthermore, infection with HCV and HBV was not treated as a time-updated variable, as this information is not available in DHCS.

## Conclusions

In a setting with free access to healthcare and antiretroviral drugs gender has no impact on time to initiation of HAART, modification of therapy the first year or response to HAART. The differences observed between genders are mainly attributable to initiation of HAART in pregnant women.

## Abbreviations

(HAART): Highly active antiretroviral therapy; (ART): Antiretroviral therapy; (ADI): AIDS defining illness; (DHCS): Danish HIV Cohort Study; (CRS): The Civil Registration System; (CPR): The Danish Personal Identification number; (HCV): Hepatitis C; (HBV): Hepatitis B; (NRTI): Nucleoside reverse transcriptase inhibitor; (NNRTI): Non-nucleoside reverse transcriptase inhibitors; (PI): Protease inhibitor; (IR): Incident rate; (IRR): Incident rate ratio; (OR): Hazard ratio; (OR): Odd ratio; (IQR): Interquartile range.

## Competing interests

KT has received honoraria from Janssen-Cilag and GlaxoSmithKline/Viiv and research funding from Abbott, TLK has received research funding from Roche, Bristol-Myers Squibb, Merck Sharp & Dohme, GlaxoSmithKline/Viiv, Abbott, Boehringer Ingelheim, Janssen-Cilag, and Swedish Orphan, NO has received research funding from Roche, Bristol-Myers Squibb, Merck Sharp & Dohme, GlaxoSmithKline, Abbott, Boehringer Ingelheim, Janssen-Cilag, and Swedish Orphan. AML has received research funding from Abbott and honoraria from Bristol-Myers Squibb, Merck Sharp & Dohme, GlaxoSmithKline, Boehringer Ingelheim and Janssen-Cilag. SL, SJF, IJ, GP and MS report no conflicts of interest.

## Authors’ contributions

KT: Analyzed and interpreted data and drafted manuscript. SL: Biostatistician; Involved in analysis and interpretation of data and provided critical review of manuscript. SJF: Contributed to conception and study design and provided critical review of manuscript. Involved in analysis and interpretation of data. ISJ: Contributed to conception and study design and provided critical review of manuscript. TLK: Contributed to conception and study design and provided critical review of manuscript. GP: Contributed to conception and study design and provided critical review of manuscript. MS: Contributed to conception and study design and provided critical review of manuscript. NO: Head of the Danish HIV Cohort Study. Contributed to conception and study design and provided critical review of manuscript. Involved in analysis and interpretation of data. AML: Principal investigator; Involved in conception and study design and provided critical review of manuscript. Involved in analysis and interpretation of data. All authors read and approved the final manuscript.

## Pre-publication history

The pre-publication history for this paper can be accessed here:

http://www.biomedcentral.com/1471-2334/12/293/prepub

## References

[B1] Mocroft A Gill MJ Davidson W Phillips AN Are there gender differences in starting protease inhibitors, HAART, and disease progression despite equal access to care? J Acquir Immune Defic Syndr 2000 24 475 482 11035619 10.1097/00126334-200008150-00013

[B2] Floridia M Giuliano M Palmisano L Vella S Gender differences in the treatment of HIV infection Pharmacol Res 2008 58 173 182 10.1016/j.phrs.2008.07.007 18708144

[B3] Kempf MC Pisu M Dumcheva A Westfall AO Kilby JM Saag MS Gender differences in discontinuation of antiretroviral treatment regimens J Acquir Immune Defic Syndr 2009 52 336 341 10.1097/QAI.0b013e3181b628be 19654551 PMC2783854

[B4] Puskas CM Forrest JI Parashar S Salters KA Cescon AM Kaida A Women and vulnerability to HAART non-adherence: a literature review of treatment adherence by gender from 2000 to 2011 Curr HIV/AIDS Rep 2011 8 277 287 10.1007/s11904-011-0098-0 21989672

[B5] Barber TJ Geretti AM Anderson J Schwenk A Phillips AN Bansi L Outcomes in the first year after initiation of first-line HAART among heterosexual men and women in the UK CHIC Study Antivir Ther 2011 16 805 814 10.3851/IMP1818 21900712

[B6] Ko NY Lai YY Liu HY Ko WC Chang CM Lee NY Gender differences in HIV manifestations at presentation to care and continuity of care among HIV-infected persons in Taiwan AIDS Care 2011 23 1254 1263 10.1080/09540121.2011.564114 21939404

[B7] http://www.europeanaidsclinicalsociety.org/index.php?option=com_content&view=article&id=59&Itemid=41. Accessed on March 26th 2012. 26-3-2012. Ref Type: Internet Communication

[B8] Soon GG Min M Struble KA Chan-Tack KM Hammerstrom T Qi K Meta-Analysis of Gender Differences in Efficacy Outcomes for HIV-Positive Subjects in Randomized Controlled Clinical Trials of Antiretroviral Therapy (2000–2008) AIDS Patient Care STDS 2012 26 444 453 10.1089/apc.2011.0278 22734949

[B9] Finocchario-Kessler S Sweat MD Dariotis JK Trent ME Kerrigan DL Keller JM Understanding high fertility desires and intentions among a sample of urban women living with HIV in the United States AIDS Behav 2010 14 1106 1114 10.1007/s10461-009-9637-8 19908135

[B10] ECDC/WHO HIV/AIDS Surveillance in Europe 2009 Available from: http://www.ecdc.europa.eu/. Accessed on June 28th 2011. 2011. Ref Type: Internet Communication

[B11] Lohse N Hansen AB Pedersen G Kronborg G Gerstoft J Sorensen HT Survival of persons with and without HIV infection in Denmark, 1995–2005 Ann Intern Med 2007 146 87 95 17227932 10.7326/0003-4819-146-2-200701160-00003

[B12] Mocroft A Ledergerber B Katlama C Kirk O Reiss P D'Arminio MA Decline in the AIDS and death rates in the EuroSIDA study: an observational study Lancet 2003 362 22 29 10.1016/S0140-6736(03)13802-0 12853195

[B13] Moore AL Sabin CA Johnson MA Phillips AN Gender and clinical outcomes after starting highly active antiretroviral treatment: a cohort study J Acquir Immune Defic Syndr 2002 29 197 202 11832692 10.1097/00042560-200202010-00015

[B14] Moore AL Kirk O Johnson AM Katlama C Blaxhult A Dietrich M Virologic, immunologic, and clinical response to highly active antiretroviral therapy: the gender issue revisited J Acquir Immune Defic Syndr 2003 32 452 461 10.1097/00126334-200304010-00017 12640206

[B15] Nicastri E Angeletti C Palmisano L Sarmati L Chiesi A Geraci A Gender differences in clinical progression of HIV-1-infected individuals during long-term highly active antiretroviral therapy AIDS 2005 19 577 583 10.1097/01.aids.0000163934.22273.06 15802976

[B16] http://www.dst.dk/da/Statistik/emner/befolkning-og-befolkningsfremskrivning/folketal.aspx. Accessed on September 14th 2012. Webpage in Danish. 14-9-2012. Ref Type: Internet Communication

[B17] http://www.ssi.dk/Service/Sygdomsleksikon/H/AIDS%20-%20HIV.aspx. Accessed on September 14th 2012. Webpage in Danish. 14-9-2012. Ref Type: Internet Communication

[B18] Petersen TS Andersen SE Gerstoft J Thorsteinsson K Larsen CS Pedersen G Adherence to national guidelines for initiation of antiretroviral regimens in HIV patients: a Danish nationwide study Br J Clin Pharmacol 2011 72 116 124 10.1111/j.1365-2125.2011.03935.x 21306418 PMC3141193

[B19] Obel N Engsig FN Rasmussen LD Larsen MV Omland LH Sorensen HT Cohort profile: the Danish HIV cohort study Int J Epidemiol 2009 38 1202 1206 10.1093/ije/dyn192 18799495

[B20] Jensen-Fangel S Pedersen L Pedersen C Larsen CS Tauris P Moller A The effect of race/ethnicity on the outcome of highly active antiretroviral therapy for human immunodeficiency virus type 1-infected patients Clin Infect Dis 2002 35 1541 1548 10.1086/344769 12471575

[B21] R Development Core Team R: A language and environment for statistical computing. R Foundation for Statistical Computing, Vienna, Austria 2011 http://www.R-project.org. 2011. Ref Type: Internet Communication

[B22] Fardet L Mary-Krause M Heard I Partisani M Costagliola D Influence of gender and HIV transmission group on initial highly active antiretroviral therapy prescription and treatment response HIV Med 2006 7 520 529 10.1111/j.1468-1293.2006.00414.x 17105511

[B23] Murri R Lepri AC Phillips AN Girardi E Nasti G Ferrara S Access to antiretroviral treatment, incidence of sustained therapy interruptions, and risk of clinical events according to sex: evidence from the I.Co.N.A. Study J Acquir Immune Defic Syndr 2003 34 184 190 10.1097/00126334-200310010-00008 14526207

[B24] Gebo KA Fleishman JA Conviser R Reilly ED Korthuis PT Moore RD Racial and gender disparities in receipt of highly active antiretroviral therapy persist in a multistate sample of HIV patients in 2001 J Acquir Immune Defic Syndr 2005 38 96 103 10.1097/00126334-200501010-00017 15608532

[B25] McNaghten AD Hanson DL Dworkin MS Jones JL Differences in prescription of antiretroviral therapy in a large cohort of HIV-infected patients J Acquir Immune Defic Syndr 2003 32 499 505 10.1097/00126334-200304150-00006 12679701

[B26] Giordano TP White AC Jr Sajja P Graviss EA Arduino RC Du-Oppong A Factors associated with the use of highly active antiretroviral therapy in patients newly entering care in an urban clinic J Acquir Immune Defic Syndr 2003 32 399 405 10.1097/00126334-200304010-00009 12640198

[B27] Stone VE HIV/AIDS in Women and Racial/Ethnic Minorities in the U.S Curr Infect Dis Rep 2012 14 53 60 10.1007/s11908-011-0226-4 22139589

[B28] Stengaard AR Lazarus JV Donoghoe MC Nielsen S Matic S Access to highly active antiretroviral therapy (HAART) for women and children in the WHO European Region 2002–2006 AIDS Care 2009 21 893 902 10.1080/09540120802537872 20024746

[B29] Obel N Omland LH Kronborg G Larsen CS Pedersen C Pedersen G Impact of non-HIV and HIV risk factors on survival in HIV-infected patients on HAART: a population-based nationwide cohort study PLoS One 2011 6 e22698 10.1371/journal.pone.0022698 21799935 PMC3143183

[B30] Thorsteinsson K Ladelund S Jensen-Fangel S Larsen MV Johansen IS Katzenstein TL Impact of gender on the risk of AIDS-defining illnesses and mortality in Danish HIV-1-infected patients: A nationwide cohort study Scand J Infect Dis 2012 10.3109/00365548.2012.684220 22803607

[B31] Hessol NA Kalinowski A Benning L Mullen J Young M Palella F Mortality among participants in the Multicenter AIDS Cohort Study and the Women's Interagency HIV Study Clin Infect Dis 2007 44 287 294 10.1086/510488 17173233

[B32] Larsen MV Omland LH Gerstoft J Larsen CS Jensen J Obel N Impact of injecting drug use on mortality in Danish HIV-infected patients: a nation-wide population-based cohort study Addiction 2010 105 529 535 10.1111/j.1360-0443.2009.02827.x 20402997

[B33] Smith C Sabin CA Lundgren JD Thiebaut R Weber R Law M Factors associated with specific causes of death amongst HIV-positive individuals in the D:A:D Study AIDS 2010 24 1537 1548 20453631 10.1097/QAD.0b013e32833a0918

[B34] Larsen MV Omland LH Gerstoft J Roge BT Larsen CS Pedersen G Impact of injecting drug use on response to highly active antiretroviral treatment in HIV-1-infected patients: a nationwide population-based cohort study Scand J Infect Dis 2010 42 917 923 10.3109/00365548.2010.511258 20840000

[B35] Collazos J Asensi V Carton JA Sex differences in the clinical, immunological and virological parameters of HIV-infected patients treated with HAART AIDS 2007 21 835 843 10.1097/QAD.0b013e3280b0774a 17415038

[B36] Zaragoza-Macias E Cosco D Nguyen ML Del RC Lennox J Predictors of success with highly active antiretroviral therapy in an antiretroviral-naive urban population AIDS Res Hum Retroviruses 2010 26 133 138 10.1089/aid.2009.0001 20156096 PMC2858896

[B37] Currier J Averitt BD Hagins D Zorrilla CD Feinberg J Ryan R Sex-based outcomes of darunavir-ritonavir therapy: a single-group trial Ann Intern Med 2010 153 349 357 20855799 10.1059/0003-4819-153-6-201009210-00002 PMC3056066

[B38] Meditz AL MaWhinney S Allshouse A Feser W Markowitz M Little S Sex, race, and geographic region influence clinical outcomes following primary HIV-1 infection J Infect Dis 2011 203 442 451 10.1093/infdis/jiq085 21245157 PMC3071223

[B39] Currier JS Spino C Grimes J Wofsy CB Katzenstein DA Hughes MD Differences between women and men in adverse events and CD4 responses to nucleoside analogue therapy for HIV infection. The Aids Clinical Trials Group 175 Team J Acquir Immune Defic Syndr 2000 24 316 324 11015147 10.1097/00126334-200008010-00003

[B40] Nightingale SL From the Food and Drug Administration JAMA 1998 280 1472 10.1001/jama.280.17.1472 9809716

[B41] De SM Carducci B De SL Cavaliere AF Straface G Periconceptional exposure to efavirenz and neural tube defects Arch Intern Med 2002 162 355 10.1001/archinte.162.3.355 11822930

[B42] Ofotokun I Chuck SK Hitti JE Antiretroviral pharmacokinetic profile: a review of sex differences Gend Med 2007 4 106 119 10.1016/S1550-8579(07)80025-8 17707845

[B43] Kesselring AM Wit FW Sabin CA Lundgren JD Gill MJ Gatell JM Risk factors for treatment-limiting toxicities in patients starting nevirapine-containing antiretroviral therapy AIDS 2009 23 1689 1699 10.1097/QAD.0b013e32832d3b54 19487907

[B44] Risk factors for lactic acidosis and severe hyperlactataemia in HIV-1-infected adults exposed to antiretroviral therapy AIDS 2007 21 2455 2464 18025882 10.1097/QAD.0b013e3282f08cdc

[B45] Andany N Raboud JM Walmsley S Diong C Rourke SB Rueda S Ethnicity and gender differences in lipodystrophy of HIV-positive individuals taking antiretroviral therapy in Ontario, Canada HIV Clin Trials 2011 12 89 103 10.1310/hct1202-89 21498152

[B46] Sanne I Mommeja-Marin H Hinkle J Bartlett JA Lederman MM Maartens G Severe hepatotoxicity associated with nevirapine use in HIV-infected subjects J Infect Dis 2005 191 825 829 10.1086/428093 15717255

[B47] Spire B Carrieri P Garzot MA L'henaff M Obadia Y Factors associated with efavirenz discontinuation in a large community-based sample of patients AIDS Care 2004 16 558 564 10.1080/09540120410001716342 15223524

[B48] Squires KE Johnson M Yang R Uy J Sheppard L Absalon J Comparative gender analysis of the efficacy and safety of atazanavir/ritonavir and lopinavir/ritonavir at 96 weeks in the CASTLE study J Antimicrob Chemother 2011 66 363 370 10.1093/jac/dkq457 21148235 PMC3019087

[B49] Falutz J Management of fat accumulation in patients with HIV infection Curr HIV/AIDS Rep 2011 8 200 208 10.1007/s11904-011-0087-3 21739217

[B50] Helleberg M Engsig FN Kronborg G Larsen CS Pedersen G Pedersen C Retention in a public healthcare system with free access to treatment: a Danish nationwide HIV cohort study AIDS 2012 26 741 748 10.1097/QAD.0b013e32834fa15e 22156974

